# Evaluation of selected ultrasonographic parameters and marker levels in the preoperative differentiation of borderline ovarian tumors and ovarian cancers

**DOI:** 10.1007/s00404-012-2453-9

**Published:** 2012-07-21

**Authors:** Piotr Sobiczewski, Anna Dańska-Bidzińska, Jakub Rzepka, Jolanta Kupryjańczyk, Mariusz Gujski, Mariusz Bidziński, Wojciech Michalski

**Affiliations:** 1 Gynecologic Oncology Department, The Maria Sklodowska-Curie Memorial Cancer Center, Medical University, 02-781 Warsaw, Poland; 2 Pathology Department, The Maria Sklodowska-Curie Memorial Cancer Center, Warsaw, Poland; 3 Department of Environment Threats and Allergology, Medical University, Warsaw, Poland; 4 Medical Center for Postgraduate Education, Warsaw, Poland; 5 Department of Biostatistic, The Maria Sklodowska-Curie Memorial Cancer Center, Warsaw, Poland

**Keywords:** Borderline ovarian tumor, Ovarian cancer, Ultrasonography, Preoperative differentiation

## Abstract

**Abstract:**

Objectives

In young patients with borderline tumors the fertility-sparing treatment is indicated, thus the preoperative investigation is important. The aim of this study was to perform a comparative assessment of sensitivity and specificity of selected ultrasonographic and clinical parameters for the diagnoses of borderline tumors and ovarian cancers.

**Methods:**

We retrospectively analyzed 57 patients who underwent surgical treatment in the Maria Sklodowska-Curie Memorial Cancer Center from Jan 01, 2008 to Dec 31, 2009. Ovarian cancers were diagnosed in 41 patients, and borderline ovarian tumors in 16 patients. Statistical model was developed to determine independent predictive factors that would be useful in preoperative differentiation between both tumors. The model included the following factors: menopausal status, tumor morphology, wall thickness (including outgrowths), septal thickness, echogenicity, resistive index, serum CA-125 level, and free fluid in the peritoneal cavity.

**Results:**

Based on the statistical model developed, independent predictive factors in the differentiation between ovarian cancers and borderline tumors included the menopausal status (*P* = 0.005), tumor echogenicity (*P* = 0.047) and the presence of free fluid in the Douglas pouch (*P* = 0.043). With the cutoff value of 13 (with scores below 13 indicating a borderline ovarian tumor, and scores of ≥13 indicating ovarian cancer), sensitivity was 90.2 % and specificity was 87 %.

**Conclusions:**

Our proposed model of preoperative evaluation has a sensitivity of 90 % in the differentiation between ovarian cancers and borderline tumors. When combined with intraoperative findings, it allows optimal surgical therapeutic decisions to be made in patients with borderline ovarian tumors.

## Introduction

Borderline ovarian tumors comprise about 15 % of all epithelial tumors of the ovary [1], and about 27 % of them occur in women below 40 years of age [2] and thus potentially willing to retain their reproductive capacity [3]. In the past, radical surgical treatment was the standard approach regardless of the patient age. Therapy included not only surgery, but also adjuvant treatment, most frequently chemotherapy.

Surgical treatment has always been a crucial component of borderline ovarian tumor therapy, and increased use of gonad-sparing or ultra-sparing surgery in the recent years has resulted in opportunities for future pregnancy in this patient group [2]. In case of women willing to retain their reproductive capacity, the decision to proceed with gonad-sparing treatment should be based on precise data that may be partially collected before the surgery.

Ultrasonography is a commonly used diagnostic tool in the evaluation of ovarian tumors. It allows rapid and noninvasive assessment of multiple parameters of tumor structure and vascular supply. Another useful marker is serum CA-125 level. In addition, patient age and family history may be helpful in estimating the risk of a malignant nature of the tumor [1].

Regarding borderline ovarian tumors, no sensitive preoperative predictive model has been yet reported in the literature. Differentiation between borderline ovarian tumors and ovarian cancers is challenging and prone to diagnostic errors. Intraoperative findings may be discordant with the final histologic diagnosis in 28–36 % of cases, and thus these patients should be managed in specialized centers [4, 5].

In this study, we attempted to develop a preoperative model to differentiate between borderline ovarian tumors and ovarian cancers based on selected ultrasonographic parameters, serum CA-125 level, and the patient menopausal status.

## Materials and methods

The purpose of the study was to compare selected ultrasonographic and clinical parameters in borderline ovarian tumors and ovarian cancers. From Jan 01, 2008 to Dec 31, 2009 eighty-eight women underwent surgical treatment for ovarian tumor at the Maria Sklodowska-Curie Memorial Cancer Center and Institute of Oncology in Warsaw, Poland. Fifty-seven patients who agreed for participation and underwent complete ultrasonographic examination were analyzed retrospectively. Ovarian cancer was diagnosed in 41 patients, and a borderline ovarian tumor was diagnosed in 16 patients.

Ultrasonographic examinations were performed by two of the authors (P.S., A.D.-B.) using the same criteria of tumor evaluation. Sonographic studies were performed preoperatively using a 7.5 MHz transvaginal probe and the Voluson 730 Expert ultrasonographic system (GR Medical Systems Kretztechnik GmbH & Co OHG) with vascular flow and three-dimensional imaging options. Tumors were examined using a power Doppler probe to visualize tumor vascular supply. When the latter was identified, the sample volume was placed in that area to measure the pulsative index (PI) and the resistance index (RI).

Tumors were categorized as unilocular cysts, multilocular cysts, solid tumors, or mixed tumors. Solid tumors were defined as tumors containing more then 80 % of solid tissue. Solid structures of >3 mm in size penetrating into the cyst lumen were classified as endophytic outgrowths. In case of different morphological structures found in the same patient, the tumor was categorized based on the most unfavorable and complex findings. The following parameters were analyzed: tumor diameter, echogenicity, the presence of outgrowths, the presence of free fluid, septal thickness, and vascular supply as assessed by PI and RI measurements.

Serum CA-125 level was determined by the immunoradiometric assay and expressed in U/mL. Increased serum CA-125 level was defined as values above 35 U/mL.

Selected tumor parameters were assigned numerical point values using a scoring system and compared between the two patient groups. The scoring system was described in Table 1.

**Table 1:** Scoring system used to evaluate selected parameters of the examined tumors

Parameter	Description	Number of points assigned
Morphology	Smooth wall	1
	Irregular wall	2
	1 endophyte	3
	>1 endophyte	4
Wall thickness	<3 mm	1
	3–5 mm	2
	>5 mm	3
Septal thickness	No septations	1
	<3 mm	2
	>3 mm	3
Echogenicity	Unilocular cyst	1
	Multilocular cyst	2
	Cyst with endophytes	3
	Mixed tumor	4
	Solid tumor	5
CA-125 level (IU/ml)^a^	<300	0
	300–500	1
	>500	2
Age	Premenopausal	1
	Postmenopausal	2
Free fluid ±	Absent	0
	Present	1
RI	<0.5	1
	>0.5	0

± the volume above 100 ml was considered abnormal

^a^CA-125 normal value <35 UI/ml, elevated value >35 UI/ml

A statistical model was developed to determine independent predictive factors that would be useful in the preoperative differentiation between ovarian cancers and borderline tumors. The model included the following factors: menopausal status, tumor morphology, wall thickness (including outgrowths), septal thickness, echogenicity, RI value, serum CA-125 level, and the presence of free fluid in the abdominal cavity and/or the rectouterine pouch.

Patient was considered as postmenopausal if she reported a period of amenorrhea of at least 12 months after the age of 40 years without other medical cause.

The stage of the disease was determined using a surgical–pathological protocol based on the International Federation of Obstetricians and Gynaecologists (FIGO) staging system for ovarian cancer. Postoperative histopathological examinations were consulted by an experienced pathologist (J.K.).

Among patients with borderline ovarian tumors, 12 operations were performed by the laparoscopic approach, and four were performed by laparotomy. A gonad-sparing surgery (cyst enucleation, adnexectomy) was performed in 13 patients, and a radical surgery was performed in three patients (Table 2).

**Table 2:** Comparison of selected clinical and histological parameters in the study groups

	Ovarian cancer	Borderline tumor	*P*
Age	68.8 (36–85)	34 (18–46)	<0.001
FIGO stage			0.017
Ia	4 (9.8 %)	4 (25 %)	
Ic	6 (14.6 %)	8 (50 %)	
IIb	1 (2.4 %)	1 (6.3 %)	
IIc	5 (12.2 %)	0	
IIIa	9 (22 %)	1 (6.3 %)	
IIIb	2 (4.8 %)	1 (6.3 %)	
IIIc	14 (34 %)	1 (6.3 %)	
Histologic type			0.015
Serous	21 (51.2 %)	14 (87.5 %)	
Mucinous	2 (4.9 %)	2 (12.5 %)	
Mixed	3 (7.3 %)	0	
Other^a^	15 (36.6 %)	0	
Type of surgery			0.01
Radical	29 (70.7 %)	3 (18.8 %)	
Sparing	0	13 (81.3 %)	
Non-radical	12 (29.3 %)	0	
Overall			
57	41	16	

^a^Endometrioid, clear cell, undifferentiated

### Statistical analysis

Standard statistical tools were used to describe the study data, including frequency tables and cross tables for categorical variables and median and extreme values for continuous variables. Statistical analysis was performed using the Statistical Package for Social Science version 15.0 (SPSS Inc., IL, USA). Descriptive statistics, Wilcoxon test, Mann–Whitney *U* test and Chi-square test were used as appropriate to obtain the presented results. Logistic regression was performed as follows: all ultrasonographic markers described in literature and available in our patients group were taken into consideration. To determine the inclusion of variables into logistic regression modeling, a *P* value of <0.1 was chosen as the critical value for statistical significance at the univariate level. All independent variables that were statistically significant at the <0.1 level in each of the univariate analyses were entered into a logistic regression analysis to determine the best predictors of tumor malignancy. Once a satisfactory model had been obtained, tests for interaction were performed on likely combinations of variables. Interaction terms were entered into the final model to determine whether a statistically significant improvement in the model was obtained.

Scoring system was based on simple punctation, where cutoff points were based on the literature available. For the CA-125 levels median values for ovarian cancer and borderline patient group were calculated. To avoid complications median values were rounded. For the purpose of the study, sensitivity, specificity, positive and negative likelihood ratios (LR+ and LR−), positive predictive value (PPV), negative predictive value (NPV), accuracy of each sonographic parameter in predicting tumor malignancy were calculated and presented with receiver operating characteristic (ROC) curves. Parameters with the highest specificity and sensitivity were included into our scoring model.

## Results

Table 2 shows the comparison of patient age, stage of disease by the FIGO classification, histologic diagnosis, and the type of surgery in the two patient groups.

In patients with ovarian cancer, the tumor diameter was increased (mean 74 mm, min. 15 mm, max. 300 mm) compared to borderline tumors (mean 62 mm, min. 31 mm, max. 210 mm; *P* = 0.044). Majority of tumors were assessed as serous histologic type: 87.5 % in borderline tumor group and 51.2 % in ovarian cancer group.

Tumor echogenicity varied between the two groups. In the ovarian cancer group, no unilocular cysts were found, a multilocular cyst was found in 8 patients (20 %), a solid tumor in 3 patients (7.5 %) and a mixed tumor with solid and cystic elements in 29 patients (72.5 %). Among patients with borderline ovarian tumors, a unilocular cyst was found in 3 patients (18 %), a multilocular cyst in 5 patients (31 %), and a tumor with endophytic outgrowths in 8 patients (50 %). No solid tumors were found in this patient group. The differences in echogenicity between the two groups were statistically significant (*P* = 0.007).

Tumor septal thickness also differed significantly between the two groups, with mean septal thickness of 6 mm in ovarian cancers compared to 3.2 mm in borderline tumors (*P* = 0.024).

The presence of free fluid in the abdominal and/or pelvic cavity was found in 21 patients with ovarian cancer (51.2 %) compared to only one patient with a borderline ovarian tumor (6.7 %; *P* = 0.003).

**Fig. 1:**
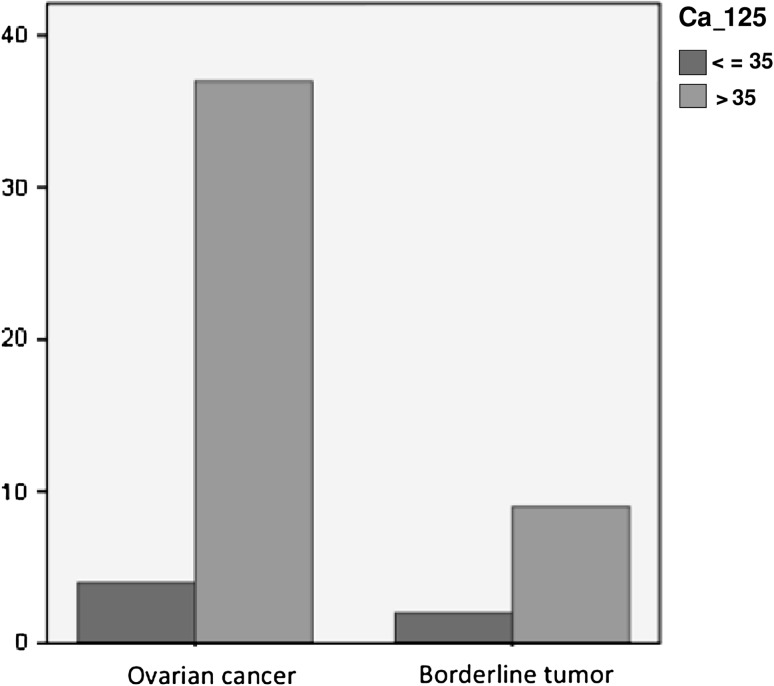
Proportion of patients with elevated (>35 UI/ml) and normal (<35 UI/ml) CA-125 levels in ovarian cancer and borderline tumor groups

Average preoperative serum CA-125 level in patients with ovarian cancer (600 U/mL) was higher compared to patients with a borderline ovarian tumor (115 U/mL; *P* = 0.004). Serum CA-125 levels in the two study groups are shown in Fig. 1.

No significant differences in RI and PI values were found between the groups. Mean RI values in ovarian cancers and borderline ovarian tumors were 0.48 and 0.51, respectively, and mean PI values were 0.94 and 0.73, respectively.

**Fig. 2:**
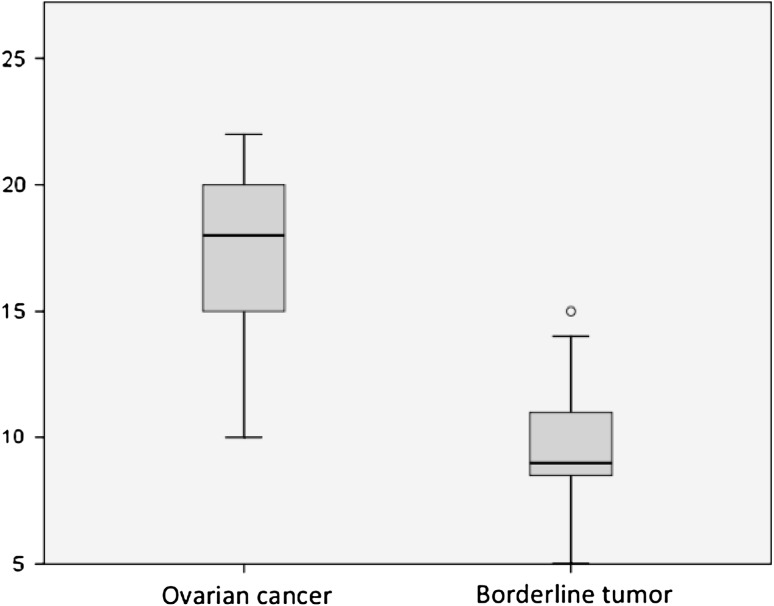
Median scores comparison in ovarian cancer vs. tumors of borderline malignancy showed with 95 % CI

**Fig. 3:**
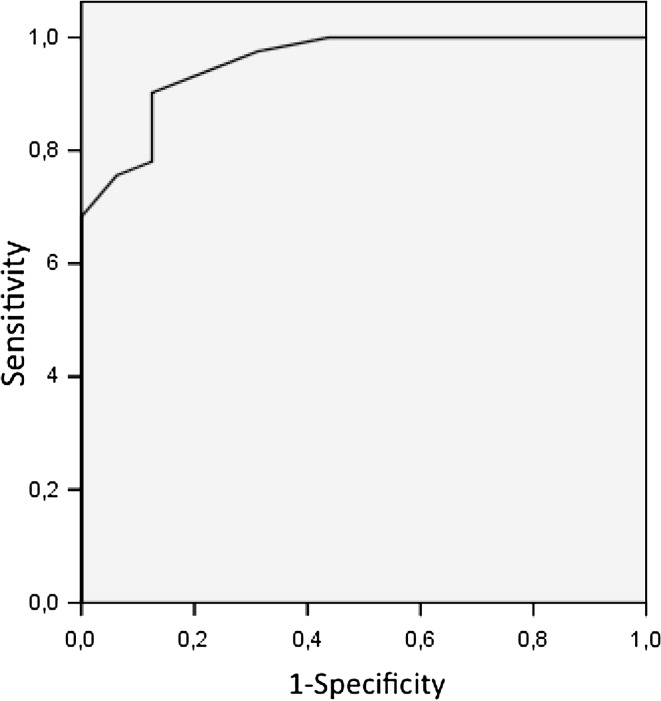
Receiver operating characteristic (ROC) curve for new scale in detection of tumor malignancy. AUC 0.955, SD 0.026, *P* < 0.001

Based on these findings and the statistical model developed, independent predictive factors in the differentiation between ovarian cancers and borderline tumors included the menopausal status (*P* = 0.005), tumor echogenicity (*P* = 0.047) and the presence of free fluid in the rectovaginal pouch (*P* = 0.043). Overall scores yielded in the two study groups in the analysis of the selected parameters with the use of our scoring system are shown in Fig. 2. The median score in the ovarian cancer group was 18 (min. 10, max. 22), compared to 9 in borderline tumors (min. 5, max. 15). We evaluated sensitivity and specificity of the test for the differentiation between ovarian cancers and borderline tumors depending on the selected cutoff value. Superior sensitivity of 90.2 % and specificity of 87 % was found for the cutoff value of 13 (with scores below 13 indicating a borderline ovarian tumor, and scores of ≥13 indicating ovarian cancer). Figure 3 shows the ROC curve illustrating the relationship between the highest achieved sensitivity and specificity of the test.

## Discussion

The diagnosis of a borderline ovarian tumor is based on histological examination, and no imaging method may reliably differentiate between borderline and malignant tumors. However, some clinical parameters, such as patient age, may suggest one or the other diagnosis, as the mean age of patients with borderline tumors is markedly younger compared to that of patients with ovarian cancer. Imaging studies, particularly ultrasonography, may be useful in the differentiation between malignant and borderline tumors when combined with other methods [6, 7]. The correct diagnosis and determining the nature of the tumor are important as prognosis is much more favorable in borderline tumors, and the latter are more often seen in younger patients in reproductive age, which affects the planned extent of the surgical treatment. Defining the ultrasonographic criteria characteristic for borderline tumors may be thus important for patients willing to preserve their reproductive capacity, as it would allow planning a gonad-sparing surgery, and in selected cases also choosing the less invasive laparoscopic approach.

In the literature, only a few studies were published comparing ultrasonographic features of borderline tumors and ovarian cancers, as most reports concerned the differentiation between benign and malignant tumors [8, 9]. In addition, borderline tumors were analyzed together with malignant tumors in many studies due to their similar morphological ultrasonographical features [10–12].

Transvaginal two-dimensional ultrasonography performed by an experienced sonographer is the most effective tool in the differentiation between benign and malignant tumors, with sensitivity of 96.7 %. Other imaging techniques, such as three-dimensional power Doppler ultrasonography, magnetic resonance imaging or even positron emission tomography do not increase sensitivity, although they may increase specificity [10, 13, 14]. One of parameters analyzed in the literature in regard to the ultrasonographic differentiation between benign and malignant tumors was the “ovarian crescent sign”. Sensitivity and specificity of the absent “ovarian crescent sign” in the differentiation between benign and malignant tumors was 94 and 40 %, respectively [11].

Morphological evaluation of the vascular supply using color Doppler and three-dimensional imaging allows evaluation of additional tumor structures but it does not increase the effectiveness of differentiating between benign and malignant tumors [10, 12, 15]. In clinical practice, a number of models based primarily on the ultrasonographic features have been introduced to facilitate this differentiation. The most commonly used tools is Risk of Malignancy Index, first described by Jacobs in 1990, which seems to have the best predictive value, but a number of other mathematical models have also been developed and await validation in the clinical practice [8, 16–20]. Combining the Lerner score and examination of vascular flow using color Doppler allows the diagnosis of a malignant tumor with a sensitivity of 92 % and a false positive rate of 19 % [9].

Studies evaluating morphological features of borderline tumors highlighted the presence of endophytic outgrowths as the most characteristic ultrasonographic finding, described as the absence of anechoic pattern and the presence of diffuse internal echoes and intracystic papillae [7]. Exacoustos et al. who reported the presence of endophytic outgrowths and multiple septations as characteristic features of borderline tumors also confirmed these observations. These authors did not find any significant differences in the ultrasonographic characteristics between specific histologic types, i.e. mucinous and serous, although the two subtypes of mucinous tumors (intestinal and endocervical or Müllerian) were combined in that study [19]. Fruscella et al. [13] compared ultrasonographic morphological features of borderline tumors of different histologic types and found differences between particular subtypes, with serous and endocervical mucinous tumors showing common features that allowed their differentiation from intestinal mucinous tumors which are associated with better outcomes. In the present study the vast majority of tumors were of serous type (87.5 %) making it impossible to draw the conclusions concerning the differences in mucinous subtypes. Valentin et al. compared morphological features of borderline and malignant tumors and found that the presence of endophytic outgrowths and multilocular cysts are characteristic for borderline tumors and stage 1 primary invasive ovarian epithelial cancers. In addition, they were less often purely solid tumors that differed from stage 2–4 ovarian epithelial cancers [6]. The prospective multicenter study proved that borderline tumors were the most difficult to correctly assess based on morphological ultrasound criteria with only 47 % being correctly classified (i.e. as malignant in this study) [21].

Color Doppler examinations indicate that a low resistance, similarly to invasive ovarian cancers, may characterize vascular flow within borderline tumors. Measurements of RI and PI yielded the values intermediate between those of benign tumors and ovarian cancers, but these differences were not statistically significant [7]. Vascular flow examination in malignant, borderline and benign tumors showed differences between these tumor types, with respective gradual lowering of RI and PI values [22]. However, these observation have not been confirmed in all studies, and reliable differentiation by color Doppler examination of vascular flow is not possible, as RI and PI values may show significant overlap between borderline and malignant tumors [14, 21].

Clinically, the diagnostic challenge is not the differentiation between malignant and benign but rather that between borderline and malignant tumors. Sensitivity of intraoperative examination in the diagnosis of borderline tumors is 60–71 % [4, 23, 24]. Thus, it seems warranted to search for criteria, by applying the available methods that would be useful in the preoperative evaluation and diagnosis. One such approach is ultrasonographic examination of morphological tumor features combined with evaluation of selected clinical parameters, which is feasible in most centers providing programs of surgical treatment of ovarian cancer.

**Fig. 4:**
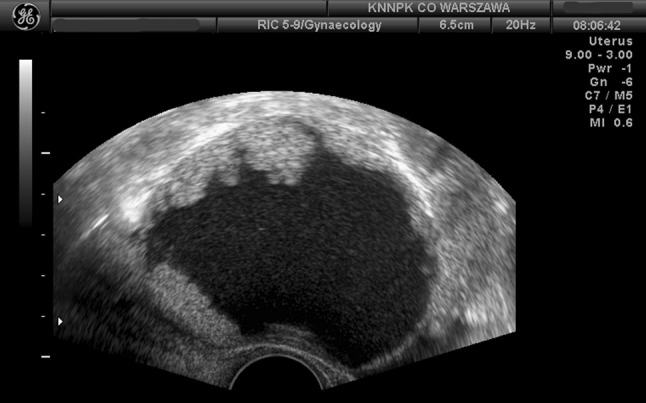
The characteristic ultrasonographic image of serous borderline tumor with typical endophytic outgrowth without free peritoneal fluid

Our scoring system including the menopausal status, tumor morphology, presence of free fluid in the pelvic cavity, and serum CA-125 level allows for better differentiation between borderline and malignant tumors. In our experience, ovarian tumors presenting in young women, characterized by the presence of endophytic outgrowths, with normal or only modestly increased CA-125 level and no free fluid in the pelvic cavity including the rectovaginal pouch and no other features of malignancy, are very likely to be borderline tumors (Fig. 4). Although definite validation of the value of this approach will require prospective studies in larger patient groups, our scoring system seems useful, as it allows preoperative identification of young patients with suspected ovarian malignancy, in which a borderline ovarian tumor is the most likely diagnosis. In case of patients willing to retain their reproductive capacity, a gonad-sparing surgery may be then planned, often performed by a minimally invasive approach. As a result, psychological aspects of preparation for the operation would also be favorably affected, reducing stress associated with the expected surgical intervention.

## Conclusions

Ultrasonographic examination before the planned surgery in patients with ovarian tumors is likely to be helpful in the differentiation between a borderline ovarian tumor and ovarian cancer.

The likelihood of ovarian cancer is very high in patients with ascites, postmenopausal women, and patients with a score of ≥13 in our clinical–morphological scoring system. In contrast, a borderline ovarian tumor is likely in young patients, premenopausal women, patients with no free fluid in the abdominal and pelvic cavity, and patients with a tumor with endophytic outgrowths and a score of <13 in our scoring system.
